# Isolation of plant growth-promoting rhizobacteria from the agricultural fields of Tattiannaram, Telangana

**DOI:** 10.1186/s43141-023-00615-5

**Published:** 2023-12-06

**Authors:** Gottumukkala Hiranmayee, Debankona Marik, Ayan Sadhukhan, Golamari Siva Reddy

**Affiliations:** 1 Department of Biotechnology, Koneru Lakshmaiah Education Foundation, Vaddeswaram, Guntur, Andhra Pradesh 522 502 India; 2 Department of Bioscience and Bioengineering, IIT Jodhpur, Jodhpur, India

**Keywords:** Plant probiotics bacteria, Phytohormones, Chemical fertilizers, Soil acidification, Nitrogen fixing, Plant immunity

## Abstract

**Background:**

Plant probiotics bacteria are live microbes that promote soil health and plant growth and build the stress-tolerant capacity to the plants. They benefit the plants by increasing nutrient absorption and release of stress-related phytohormones. These plant probiotic bacteria serve a better purpose to the plant when compared to chemical fertilizers. Use of chemical fertilizers such as arsenic and cadmium can lead to soil acidification and even release of harmful gases such as methane which further pollutes the environment.

**Results:**

Different bacterial species were isolated from the agricultural fields of Tattiannaram, Telangana, and identified as the efficient rhizosphere bacteria with the essential qualities of plant growth promotion by evaluating the nitrogen-fixing ability on a selective media and various other methods. Upon the molecular characterization of the isolates, they were identified as *Corynebacterium* spp., *Bacillus* spp., *Lactobacillus* spp., and *Cytobacillus* spp. The results were also examined using various bioinformatics tools for accuracy in their phylogenetic pattern.

**Conclusion:**

The recognized species of plant probiotics have established roles in promoting plant growth and strengthening plant immunity. This research introduces an innovative methodology for evaluating and investigating recently identified bacterial isolates, focusing on their distinctive plant probiotic attributes. Through harnessing the potential of advantageous microorganisms and comprehending their interaction with plants and soil, our objective is to formulate inventive approaches to elevate crop productivity, enhance soil richness, and foster environmentally sustainable and robust agricultural methodologies. These characteristics exhibit promising potential for future incorporation into plant systems, fortifying growth and development, and underscoring their distinctive significance within the realm of agriculture.

**Supplementary Information:**

The online version contains supplementary material available at 10.1186/s43141-023-00615-5.

## Background

Soil, the organic geological skin of the earth, is essential for life and habitat to an incredible microbial community. Microbes have mostly been regarded as stewards of the biosphere [1]. Soil bacteria are global drivers of critical biogeochemical cycles including carbon, nitrogen, phosphorus, iron, and other elements. Their process supports a wide range of soil physiological functions, such as the cycling of nutrients and the establishment of soil structure [2]. Soil is granular, discontinuous, and patchy, with many geographically unique microenvironments associated with various physicochemical conditions coexisting to create a diverse range of microbial homes [3, 4]. One of the main important features of the soil is to aid the growth of diversified living creatures. One such important living body is a plant. Plants need soil as a substrate because it provides them with anchoring, as well as water and nutrients for phototrophic development. Plant roots account for one-third of all phytobiomass on the globe [5]. The plant roots penetrate the soil actively seeking nutrients [6, 7]. Plant roots are home to a variety of microorganisms that provide a variety of functions that are necessary for plant growth. Many species aid in the mineralization of nutrients, the production of growth hormones, and the prevention of illnesses [8, 9]. Microorganisms are important in plant development, nutrition management, and biocontrol activities. These microbes invade plant rhizosphere and stimulate plant development via various cellular processes [10]. Plant–microbe interactions are the best examples of mutualistic association in the ecosystem [11]. Even though numerous roles have been identified, harnessing the microbial communities has proved impossible. This is especially true in soils that have been damaged by intensive agriculture. Low microbial biodiversity in such soils limits the number of plant-biological activities and renders microorganisms’ expression of those functions unreliable [12]. As a result, restoring microbial biodiversity may increase soil fertility, re-establishing community-level acquisition of the various plant-beneficial services required for strong development [13, 14].

Plants interact with a range of microbes in both aerial and subsoil tissues, establishing associations that can be harmful (pathogenic), neutral, or advantageous to the host plant. Several studies have shown evidence of the ability of microbes to boost plant development in various cultivated species. One such criterion is the development of plant growth-promoting bacteria. Plant growth-promoting bacteria (PGPB) are one of the most generalized findings of plant probiotic microbes (PPM) in soil, having *Bacilli* and *Pseudomonas* being the most common genera reported [15–17]. Plant growth-promoting abilities are seen in both rhizosphere and endophytic soil bacteria. Rhizosphere microorganisms are often located in the root system, whereas endophytic bacteria are either distributed within the plant’s tissues or are available as free-living soil microorganisms [18]. Upon consideration of its vital role, plant growth-promoting rhizobacteria (PGPR) has evolved as a founder association between plants and microbes, expressing antagonistic and synergistic responses with bacteria and the soil. Bio-fertilization, revitalizing plant development, rhizo-remediation, resistance to disease, and other approaches of microbial rejuvenation involving plant growth stimulants have been used [19]. Several research has emphasized plant probiotics as a potential soil agronomic source; their application in agriculture enhances nutrient availability, preserving the natural field maintenance, and has no negative consequences [20].

Probiotics are living microbial species utilized to improve the health and vitality of the host. Microorganisms employed for probiotic purposes can be found in nature, but they must be correctly identified, isolated, and studied for virulence [21]. Plant probiotics, like human probiotics, are microbial cultures with plant improvement and biocontrol ability. Plant probiotics help in nitrogen fixation, phosphate solubilization, siderophores synthesis, and disease resistance [22–24]. For example, the wilt of banana disease caused by *Fusarium oxysporum* is treated by *Bacillus subtilis*ssp [25].. Plant probiotics include bacteria engaged in phosphate solubilization, nitrogen fixation, and biological disease control [26, 27].

Haas and Keel coined the term plant probiotic bacteria (PPB) to describe a group of microorganisms that benefit plants and meet three necessary attributes that when combined result in improved plant protection: (i) niche colonization effectiveness and competitiveness, (ii) ability to induce systemic resistance (ISR) in their hosts, and (iii) existence of specific antagonistic traits on pathogens [28].

Agriculture and forestry are constantly being impacted by increasing population, soil degradation, environmental degradation, and global warming [29]. This has led to the development of PPM-based technologies as a replacement for bio-stimulants, biocontrol agents, and environmental remediation [30]. New agroecological approaches are being made by utilizing existing natural resources [31]. Plant probiotic bacteria promote host plant development, especially yield, and may also prevent diseases when used in sufficient quantities [32].

In long-term agricultural production, nitrogen fertilizers are frequently used to boost early crop output. Long-term administration of nitrogen fertilizers, on the other hand, can alter the plant soil microbe system by altering the composition of vegetation and soil microbial populations [33–36]. Nitrogen (N) availability is one of the major variables limiting terrestrial ecosystem production. Nitrogen fixation is a magnificent phenomenon responsible for almost two-thirds of all nitrogen fixation on the planet. The symbiotic or nonsymbiotic interactions between bacteria and plants carry out this biological activity [37]. As a result, restoring microbial biodiversity may increase soil fertility, re-establishing community-level acquisition of the various plant-beneficial services required for strong development. This research introduces a fresh pathway for the screening and profiling of novel bacterial isolates, focusing on their probiotic attributes. These attributes hold promise for prospective implementation in plant systems to enhance growth support.

## Methods

**Fig. 1:**
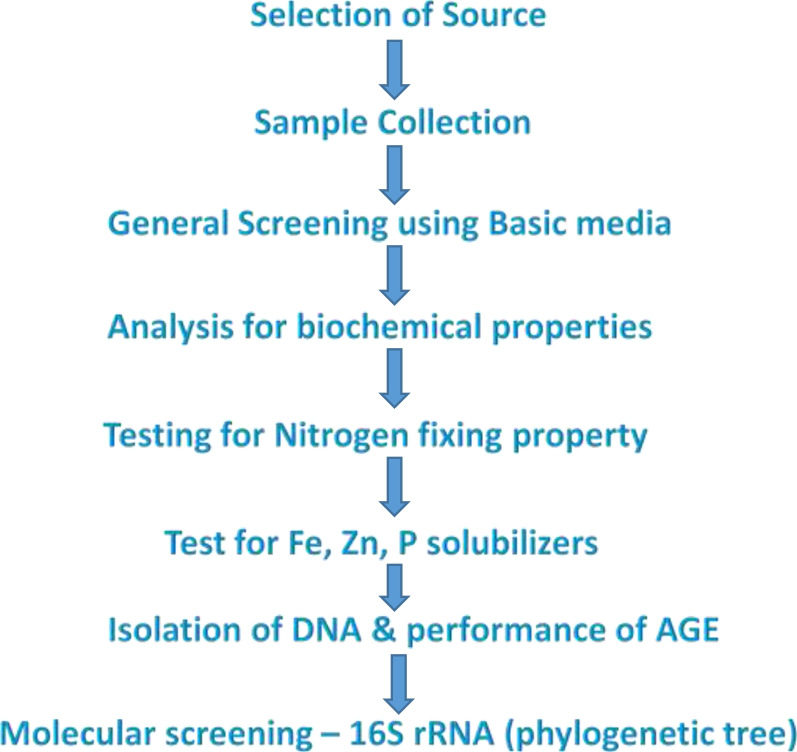
Flowchart for the methodology

The entire methodology followed during the work is represented in Fig. 1.

### Sample collection

**Fig. 2:**
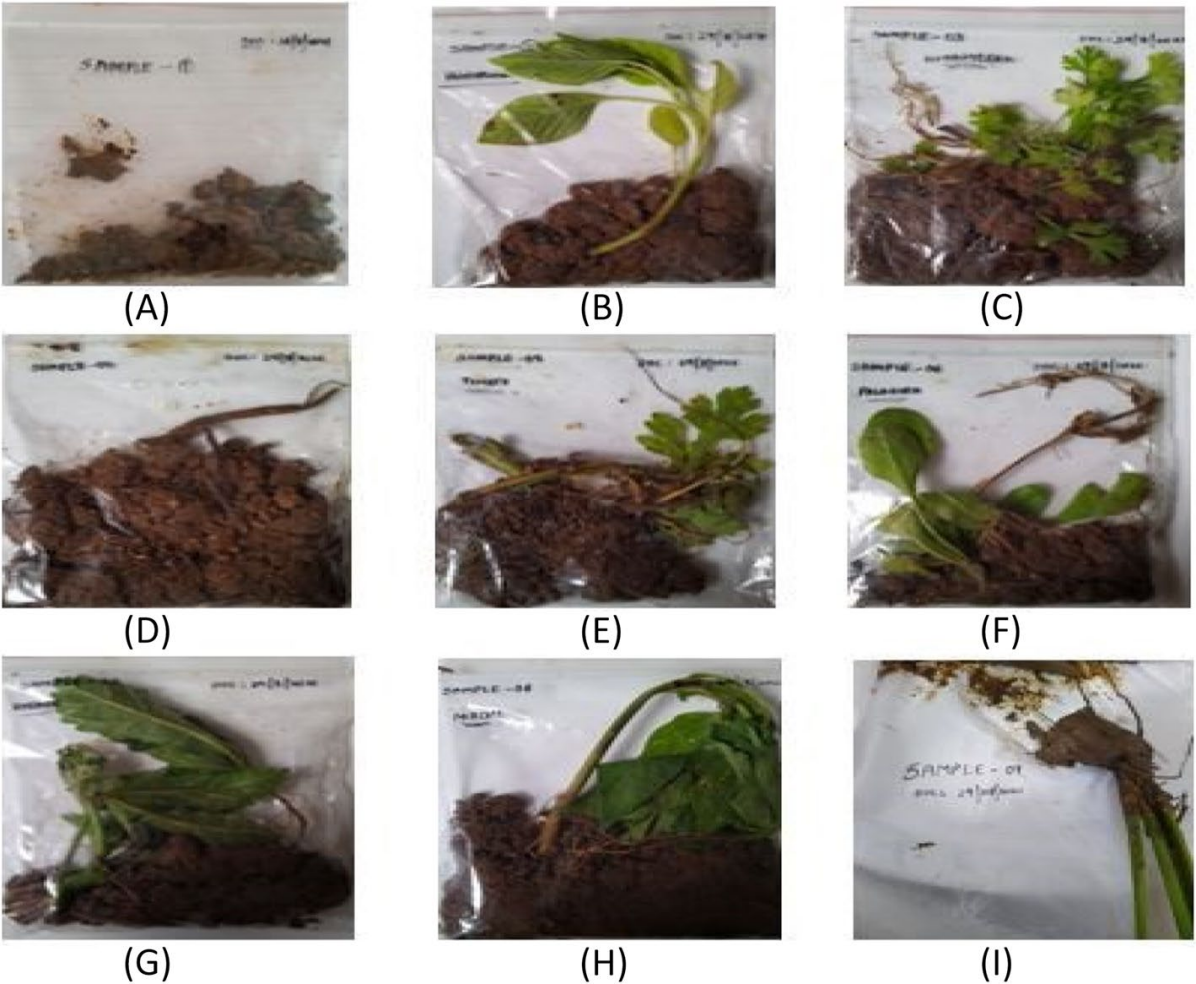
Soil samples collected from the rhizosphere of various plants. **A** *Ocimum sativum*. **B** *Amaranthus viridis*. **C** *Coriandrum sativum*. **D** *Solanum melongena*. **E** *Solanum lycopersicum*. **F** *Spinacia oleracea*. **G** *Abelmoschus esculentus*. **H** *Capsicum annum*. **I** *Oryza sativa*

Soil samples were gathered from the Tattiannaram fields in Telangana, India (Fig. 2). Tattiannaram is an agricultural region situated in Telangana, characterized by a moderate climate and a diverse array of soil microorganisms. To account for various factors such as soil type, topography, and crop history, the land was divided into distinct zones. The rhizosphere zone of the plants was the focus for soil collection, spanning from 0 to 15 cm below the surface, aimed at conducting microbial analysis. To ensure unbiased and comprehensive representation, a random sampling approach was employed. The collected samples were immediately placed in sterile sealable bags, each containing 100 g of soil. This meticulous process guarantees the integrity of the collected samples and their suitability for further analysis.

### Preliminary screening

**Fig. 3:**
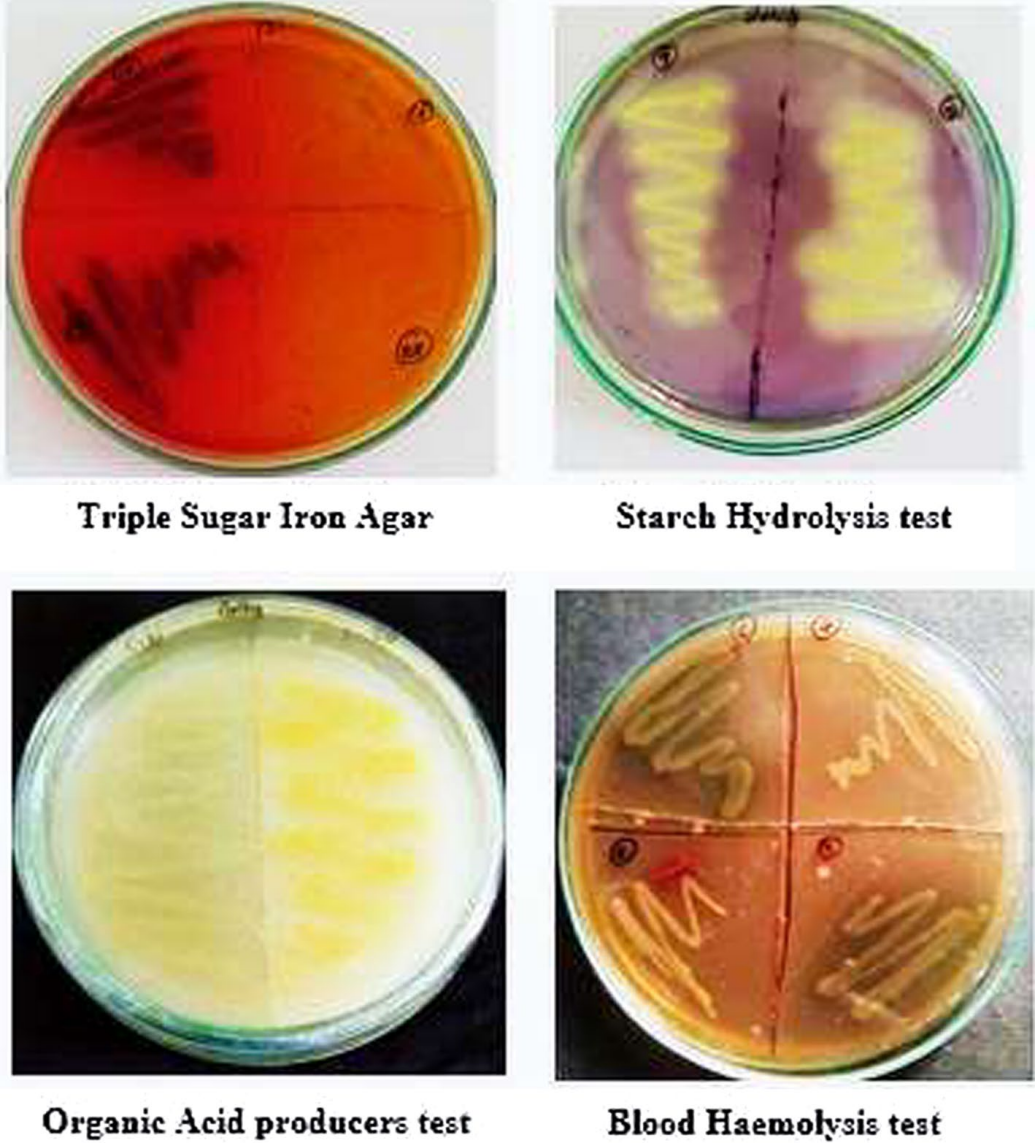
Microbial activity on triple sugar iron agar, starch agar, organic acid, and blood agar

The rhizosphere is considered an important plant nutrient source where most of the microbes make up this as a resident club. The first step in developing a producer strain is the isolation of concerned microorganisms from their natural habitats. A set of highly selective procedures which allows the detection and isolation of microbes producing the desired metabolite constitute primary screening. However, this is possibly the most critical step since it eliminates the bulk of unwanted useless isolates, either nonproducers or producers of known compounds. As a part of the primary screening technique, the isolation of the bacteria was done using basic lab equipment and certain media. To analyze for the various microbes within the collected soils, the basic medium such as nutrient agar was used, believing that majority of microbes can be screened. Each soil sample was serially diluted and plated as per the standard protocols of microbial isolation techniques. After the growth observed on basic media, the isolates with different morphological features were subcultured and further tested for biochemical properties. The biochemical properties were analyzed by performing IMViC tests, catalase, coagulase, oxidase, starch hydrolysis, organic acid production tests and blood haemolysis test, etc. In detail, the IMViC test helps to narrow down the group of bacteria and provide information about their metabolic capabilities. Plant probiotic bacteria often produce organic acids through their metabolic processes. These organic acids can help in solubilizing minerals, making them more available to plants. The ability to produce organic acids like citric acid and malic acid can be a desirable trait in plant probiotic bacteria. The purpose of performing various biochemical tests was tabulated in Table 1. The isolates were inoculated on starch agar, TSI, urease, and blood agar which were depicted in Fig. 3. Bacteria with amylase activity can potentially contribute to nutrient availability for plants by breaking down starch present in the soil or plant debris. The urease test helps to identify bacteria capable of producing urease. Such bacteria can increase the availability of nitrogen in the soil by converting urea into ammonia, which can be utilized by plants as a nitrogen source. In the context of plant probiotics, the TSI test can provide insights into the bacterial metabolism of sugars and gas production. Gas production is relevant as it can influence soil structure and aeration, and hydrogen sulfide production can also impact plant growth. The blood agar test might provide the insights into bacterial interactions with organic matter and potential nutrient release which corresponds to plant probiotic activity. Subsequently, selected isolates were screened by maintaining them as liquid cultures using a shaker. The isolates with similar properties including morphology and staining features were eliminated.

**Table 1:** Test information and results

Test	Purpose of the test	Validation of the result	
		**Positive**	**Negative**
Indole test	Screen for the organism that degrades tryptophan and produces indole	Cherry red ring formation	Yellow/no ring formation
Methyl red	Detects if the organism performs mixed acid fermentation	Red	Yellow
Voges Proskauer	Detects the presence of acetoin within the metabolic pathway	Red/purple color	No color
Citrate utilization	Screen for the organism that uses citrate as carbon and energy source	Blue	Green
Catalase	Used for the detection of enzyme catalase	Effervescence	No effervescence
Coagulase	Used for the detection of protein coagulase	Clot formation	No clot
Oxidase	Assay for the presence of cytochrome oxidase	Purple	Yellow/no color
Urease	To test the ability of microbe that degrades urea	Pink	Yellow
Triple sugar iron agar	Determination of carbohydrate fermentation and H_2_S production	Pink with gas	Yellow
Blood hemolysis	Test for the ability of organism that produces haemolysin	Growth seen	No growth
Starch hydrolysis	Test for the ability of organism that hydrolyses starch	Blue with zone of inhibition	No zone
Organic acid production	Detection of microbe if it produces any organic acid as an intermediate	Thick white colony growth	Pale /transparent growth

Later, the studies were carried out to screen out the nitrogen-fixing property using certain specific media such as Jensen’s medium (sucrose 2%, K_2_PO_4_ 0.1%, MgSO_4_.7H_2_O 0.05%, NaCl 0.05%, FeSO_4_ 0.01%, Na_2_Mo 0.0005%, CaCO_3_ 0.2%, agar 1.5%). First, the isolates were inoculated on solid medium. Later to estimate their growth levels, the isolates were re-examined on liquid medium. This growth was analyzed for 24 h and 48 h by colorimetric analysis at 600 nm. Bacteria that thrive on Jensen’s media might have the ability to fix nitrogen, making them potentially beneficial for plants. The organisms that have shown positive with Jensen’s media were further examined if they can also utilize and tolerate to different chemical exposures such as zinc, phosphorus, and iron using modified zinc solubilizing medium (NaCl 0.468%, NH4Cl 0.107%, MgSO_4_.7H_2_O 0.043%, CaCl_2_.2H_2_O 0.003%, ZnSO_4_ 0.01%, agar 1.5%, pH 6.6 ± 0.2), modified phosphate-solubilizing medium (yeast extract 0.05%, dextrose 1%, Ca_2_PO_3_ 0.5%, NH_4_(SO4)_2_ 0.02%, MgSO_4_.7H_2_O 0.010%, FeSO_4_ 0.00001%, pH 7), and modified iron-solubilizing media (FeCl_3_ 0.1%, peptone 1%, casein 1%, tryptone 0.5%, NaCl 1%, glucose 1%, agar 1.5%, pH 7.1 ± 0.2), respectively. The growth rate was analyzed by measuring the OD (optical density value) which is set to 540 nm (nanometers) and 580 nm using a colorimeter. Plants often face challenges in accessing essential nutrients like zinc, iron, and phosphorus due to their limited solubility in the soil. Bacteria capable of solubilizing these minerals can enhance nutrient availability for plants. The organisms which have shown good growth rate and minimum standard deviation upon all the triplicate values were selected as the best isolates and moved for further examination. The selected isolates were inoculated to Ashby’s liquid medium (KH_2_PO_4_ 0.02%, CaCO_3_ 0.5%, MgSO_4_.7H_2_O 0.02%, mannitol 1%, NaCl 0.02%, CaSO_4_.2H_2_O 0.01%, agar 1.5%) to confirm the nitrogen-fixing property [38] and compare them with Jensen’s medium results. The purpose of performing these tests helps in narrowing down the possibilities and focusing on strains that exhibit the desired traits for plant growth promotion, such as nutrient solubilization, nitrogen fixation, and organic acid production.

### Molecular identification

After the initial screening of the isolates, a subsequent molecular screening process was conducted. This involved extracting DNA using the Bio-Pure™ kit from BioAxis DNA Research Centre located in Hyderabad. Following this, the PCR technique was applied to amplify a specific gene from the extracted DNA samples. This gene amplification was achieved using the 27f and U1492r primers with the sequences 5′-AGAGTTTGATCCTGGCTCAG-3′ and 5′-TACGGTTACCTTGTTACGACTT-3′, respectively [39]. Following the gene amplification, forward and reverse sequencing reactions were carried out on an ABI 3730XL sequencer to obtain the sequence data. The separation method used for this process involved agarose gel electrophoresis technique (AGE) utilizing a 1% agarose gel. This method is fundamental for separating and analyzing DNA fragments or RNA molecules based on their respective sizes. The basis of this technique is the inherent negative charge carried by nucleic acids. To implement this method, a 1000 base pair (bp) amplicon and a 1-kb molecular ladder were used. Consequently, a mixture of DNA or RNA fragments was distinctly separated into bands along the length of the gel, with each band indicating a fragment of a unique size.

The acquired 16S rRNA sequence serves as the basis for discerning analogous sequences and potential taxonomic associations through the utilization of BLAST analysis. The procedure involves accessing the NCBI BLAST website, opting for a relevant database (such as “16S ribosomal RNA sequences (bacteria and archaea)”), inputting the 16S rRNA sequence into the query box, adjusting parameters, and subsequently initiating the BLAST search [40]. Subsequent to the search, the outcomes of the BLAST analysis are meticulously reviewed to pinpoint the closest matches and their corresponding taxonomic details. Subsequently, the ten most similar sequences are transferred to the Clustal Omega tool, where the aligned sequences undergo scrutiny to detect conserved segments and discrepancies, offering insights into the underlying phylogenetic relationships [41]. This process culminates in the construction of a cladogram that visually depicts the percentile similarity among the sequences.

Later, the bootstrapping technique was employed as it is crucial for making well-informed evolutionary interpretations, as it addresses the inherent noise in genetic data and the complexities of evolutionary processes [42]. Using the 1000 bootstrap alignments and the 1000 iterations, the phylogenetic tree of the sequence dataset is constructed using IQ-TREE server, and for enhancing the reliability of the predicted phylogenetic solution, the minimum correlation coefficient of 0.99 is used.

## Results

Upon screening the isolates by considering the basic traits, a total of 101 isolates were found initially. Colony morphological testing, gram staining, and biochemical tests (IMViC, catalase, coagulase, oxidase, TSI, urease, and hemolysis tests) were performed. The organisms with similar traits were re-examined and eliminated. For the second stage of screening, 36 isolates were selected. The selected isolates were highlighted in Table 2, and those isolates were further tested for properties such as amylase production by starch hydrolysis and screened for the organic acid production using calcium carbonate media (Table 3).

**Table 2:** Screening of bacterial isolates by preliminary tests

S/no	Source plant	Isolate names	Indole	MR	Vp	Citrate	Catalase	Oxidase	Urease	TSI	Blood hemolysis
**1**	***Ocimum sativum***	KL-001	** + **	**-**	**-**	**-**	**-**	**-**	** + **	**-**	**-**
		KL-002	**-**	** + **	** + **	** + **	**-**	**-**	** + **	**-**	**-**
		KL-003	**-**	** + **	**-**	**-**	** + **	** + **	**-**		**-**
		**KL-004**	**-**	**-**	**-**	** + **	** + **	**-**	**-**	** + **	**-**
		**KL-005**	**-**	**-**	**-**	**-**	**-**	**-**	** + **	**-**	**-**
		KL-006	**-**	** + **	** + **	**-**	**-**	**-**	**-**	**-**	** + **
		KL-007	**-**	** + **	** + **	** + **	** + **	**-**	**-**	** + **	**-**
		KL-008	**-**	**-**	**-**	** + **	** + **	**-**	**-**	** + **	**-**
		KL-009	**-**	** + **	**-**	** + **	** + **	**-**	**-**	**-**	**-**
		**KL-010**	**-**	**-**	**-**	**-**	** + **	**-**	**-**	**-**	**-**
		**KL-011**	**-**	** + **	**-**	** + **	** + **	**-**	**-**	** + **	**-**
		KL-012	**-**	** + **	**-**	**-**	** + **	** + **	**-**		**-**
		KL-013	** + **	**-**	**-**	**-**	**-**	**-**	** + **	**-**	**-**
		**KL-014**	**-**	** + **	** + **	** + **	** + **	**-**	**-**	** + **	**-**
		**KL-015**	**-**	**-**	** + **	** + **	** + **	**-/ + **	**-**	**-**	**-**
		KL-016	**-**	** + **	** + **	** + **	** + **	** + **	**-**	**-**	**-**
		KL-017	**-**	** + **	**-**	**-**	** + **	** + **	**-**		**-**
		KL-018	** + **	**-**	**-**	**-**	**-**	**-**	** + **	**-**	**-**
		KL-019	**-**	** + **	** + **	** + **	** + **	** + **	**-**	**-**	**-**
		KL-020	**-**	** + **	**-**	** + **	** + **	**-**	**-**	**-**	**-**
		KL-021	**-**	**-**	**-**	** + **	** + **	**-**	**-**	** + **	**-**
		**KL-022**	**-**	** + **	**-**	** + **	** + **	**-**	**-**	**-**	**-**
		**KL-023**	** + **	** + **	**-**	** + **	**-**	**-**	**-**	** + **	**-**
		KL-024	** + **	** + **	**-**	** + **	**-**	**-**	**-**	** + **	**-**
		KL-025	** + **	**-**	**-**	**-**	**-**	**-**	** + **	**-**	**-**
		KL-026	**-**	** + **	** + **	** + **	** + **	**-**	**-**	**-**	**-**
		KL-027	**-**	**-**	**-**	** + **	** + **	**-**	**-**	** + **	**-**
		KL-028	** + **	** + **	** + **	** + **	**-**	**-**	**-**	**-**	**-**
		KL-029	** + **	** + **	**-**	** + **	**-**	**-**	**-**	** + **	**-**
		KL-030	**-**	** + **	** + **	** + **	** + **	** + **	**-**	**-**	**-**
		**KL-031**	**-**	** + **	** + **	** + **	** + **	** + **	**-**	** + **	**-**
		**KL-032**	**-**	** + **	**-**	** + **	** + **	**-**	**-**	**-**	**-**
		KL-033	**-**	** + **	** + **	** + **	** + **	**-**	**-**	**-**	**-**
**2**	***Amaranthus viridis***	KL-034	**-**	** + **	** + **	** + **	** + **	**-**	**-**	**-**	**-**
		KL-035	**-**	** + **	** + **	** + **	** + **	** + **	**-**	**-**	**-**
		KL-036	**-**	**-**	** + **	** + **	** + **	** + **	**-**	**-**	**-**
		KL-037	** + **	** + **	**-**	** + **	**-**	**-**	**-**	** + **	**-**
		KL-038	**-**	** + **	**-**	** + **	** + **	**-**	**-**	** + **	**-**
		KL-039	**-**	** + **	**-**	** + **	** + **	** + **	**-**	** + **	**-**
		**KL-040**	**-**	** + **	**-**	** + **	** + **	**-**	**-**	**-**	**-**
		**KL-041**	**-**	** + **	** + **	** + **	** + **	**-**	**-**	**-**	**-**
		KL-042	**-**	** + **	**-**	** + **	** + **	** + **	**-**	** + **	**-**
		KL-043	**-**	** + **	**-**	**-**	**-**	**-**	**-**	**-**	**-**
		**KL-044**	**-**	** + **	**-**	** + **	** + **	**-**	**-**	** + **	**-**
		**KL-045**	**-**	**-**	** + **	** + **	** + **	** + **	**-**	**-**	**-**
**3**	***Coriandrum sativum***	KL-046	**-**	** + **	**-**	** + **	** + **	** + **	**-**	** + **	**-**
		KL-047	**-**	**-**	**-**	** + **	-	-	**-**	** + **	**-**
		**KL-048**	**-**	** + **	**-**	** + **	**-**	**-**	**-**	**-**	**-**
		KL-049	**-**	**-**	**-**	** + **	-	-	**-**	** + **	**-**
		KL-050	**-**	** + **	**-**	** + **	** + **	** + **	**-**	** + **	**-**
		KL-051	**-**	** + **	**-**	** + **	** + **	** + **	**-**	** + **	**-**
		**KL-052**	**-**	** + **	**-**	** + **	** + **	** + **	**-**	** + **	**-**
		**KL-053**	** + **	** + **	**-**	** + **	** + **	** + **	**-**	** + **	**-**
		KL-054	** + **	**-**	**-**	**-**	**-**	**-**	**-**		**-**
		**KL-055**	**-**	** + **	**-**	** + **	** + **	** + **	**-**	** + **	**-**
**4**	***Solanum melongena***	KL-056	** + **	** + **	**-**	** + **	** + **	**-**	**-**	** + **	**-**
		KL-057	** + **	** + **	**-**	** + **	** + **	**-**	**-**	** + **	**-**
		KL-058	**-**	** + **	**-**	** + **	** + **	** + **	**-**	** + **	**-**
		KL-059	** + **	** + **	**-**	** + **	** + **	** + **	**-**	** + **	**-**
		KL-060	**-**	** + **	**-**	** + **	** + **	** + **	**-**	** + **	**-**
		KL-061	**-**	** + **	**-**	** + **	** + **	** + **	**-**	** + **	**-**
		KL-062	** + **	**-**	**-**	**-**	**-**	**-**	** + **	**-**	**-**
**5**	***Solanum lycopersicum***	KL-063	**-**	** + **	** + **	** + **	**-**	**-**	** + **	**-**	**-**
		KL-064	**-**	** + **	**-**	**-**	** + **	** + **	**-**		**-**
		**KL-065**	**-**	** + **	**-**	** + **	** + **	** + **	**-**	**-**	**-**
		KL-066	** + **	** + **	**-**	** + **	** + **	**-**	**-**	** + **	**-**
		KL-067	**-**	** + **	**-**	** + **	** + **	** + **	**-**	** + **	**-**
		KL-068	** + **	** + **	**-**	** + **	** + **	**-**	**-**	** + **	**-**
		**KL-069**	** + **	** + **	**-**	** + **	** + **	**-**	**-**	** + **	**-**
		KL-070	** + **	** + **	**-**	**-**	**-**	**-**	**-**	**-**	**-**
		**KL-071**	** + **	** + **	**-**	** + **	** + **	**-**	**-**	** + **	**-**
**6**	***Spinacia oleracea***	KL-072	** + **	** + **	**-**	**-**	**-**	**-**	**-**	**-**	**-**
		**KL-073**	** + **	** + **	** + **	**-**	**-**	** + **	**-**	**-**	**-**
		KL-074	** + **	** + **	**-**	** + **	** + **	**-**	**-**	** + **	**-**
		KL-075	**-**	** + **	**-**	** + **	** + **	** + **	**-**	** + **	**-**
		**KL-076**	**-**	**-**	**-**	**-**	**-**	**-/ + **	**-**	**-**	**-**
		**KL-077**	** + **	** + **	**-**	**-**	**-**	**-**	**-**	**-**	**-**
		**KL-078**	** + **	** + **	**-**	** + **	** + **	** + **	**-**	**-**	**-**
		**KL-079**	** + **	** + **	**-**	** + **	**-**	**-**	**-**	** + **	**-**
		**KL-080**	** + **	** + **	**-**	** + **	** + **	**-**	**-**	** + **	**-**
**7**	***Abelmoschus esculentum***	**KL-081**	**-**	** + **	**-**	**-**	** + **	**-**	**-**	** + **	**-**
		KL-082	**-**	** + **	** + **	**-**	**-**	**-**	**-**	**-**	**-**
		**KL-083**	** + **	**-**	**-**	** + **	** + **	** + **	**-**	** + **	**-**
		KL-084	** + **	** + **	**-**	** + **	** + **	**-**	**-**	** + **	**-**
		KL-085	**-**	** + **	**-**	** + **	** + **	** + **	**-**	** + **	**-**
**8**	***Capsicum annuum***	KL-086	** + **	** + **	**-**	** + **	** + **	**-**	**-**	** + **	**-**
		**KL-087**	** + **	** + **	**-**	** + **	**-**	** + **	**-**	**-**	**-**
		**KL-088**	** + **	** + **	** + **	**-**	**-**	** + **	**-**	**-**	**-**
		**KL-089**	**-**	** + **	**-**	**-**	** + **	** + **	**-**	** + **	**-**
		KL-090	**-**	** + **	**-**	** + **	** + **	** + **	**-**	** + **	**-**
		**KL-091**	** + **	** + **	**-**	** + **	** + **	** + **	**-**	** + **	**-**
		KL-092	**-**	** + **	**-**	** + **	** + **	** + **	**-**	** + **	**-**
**9**	***Oryza sativa***	**KL-093**	** + **	** + **	**-**	**-**	** + **	** + **	**-**	**-**	**-**
		**KL-094**	** + **	** + **	** + **	** + **	** + **	** + **	** + **	**-**	**-**
		KL-095	** + **	** + **	**-**	** + **	** + **	**-**	**-**	** + **	**-**
		KL-096	**-**	** + **	**-**	** + **	** + **	** + **	**-**	** + **	**-**
		KL-097	** + **	** + **	**-**	** + **	** + **	**-**	**-**	** + **	**-**
		**KL-098**	** + **	** + **	** + **	** + **	** + **	** + **	** + **	**-**	**-**
		KL-099	**-**	** + **	**-**	** + **	** + **	** + **	**-**	** + **	**-**
		KL-100	** + **	** + **	**-**	** + **	** + **	** + **	**-**	** + **	**-**
		KL-101	** + **	** + **	**-**	** + **	** + **	**-**	**-**	** + **	**-**

( +) indicates results were positive; (-) indicates results were negative

**Table 3:** Determination of organic acid production and starch hydrolytic abilities of isolated bacteria

Isolate number	Starch hydrolysis	Organic acid producers
KL-014	**-**	** + **
KL-015	** + **	** + **
KL-011	**-/ + **	** + **
KL-010	** + **	** + **
KL-022	** + **	** + **
KL-023	** + **	** + **
KL-044	**-**	** + **
KL-045	** + **	** + **
KL-040	** + **	** + **
KL-041	** + **	** + **
KL-048	** + **	**-**
KL-052	**-**	**-**
KL-053	** + **	** + **
KL-055	** + **	**-**
KL-065	**-**	**-**
KL-069	** + **	**-**
KL-071	**-**	**-**
KL-073	**-**	**-**
KL-076	**-**	**-**
KL-077	**-**	**-**
KL-078	**-**	**-**
KL-080	**-**	** + **
KL-079	** + **	** + **
KL-081	** + **	**-**
KL-083	**-**	**-**
KL-087	** + **	**-**
KL-088	** + **	** + **
KL-089	** + **	** + **
KL-091	** + **	** + **
KL-094	** + **	** + **
KL-098	**Slightly ( +)**	** + **
KL-093	**-**	**-**
KL-031	**-**	**-**
KL-032	**-**	** + **
KL-005	** + **	** + **
KL-004	**-**	**-**

( +) indicates results were positive; (-) indicates results were negative

**Fig. 4:**
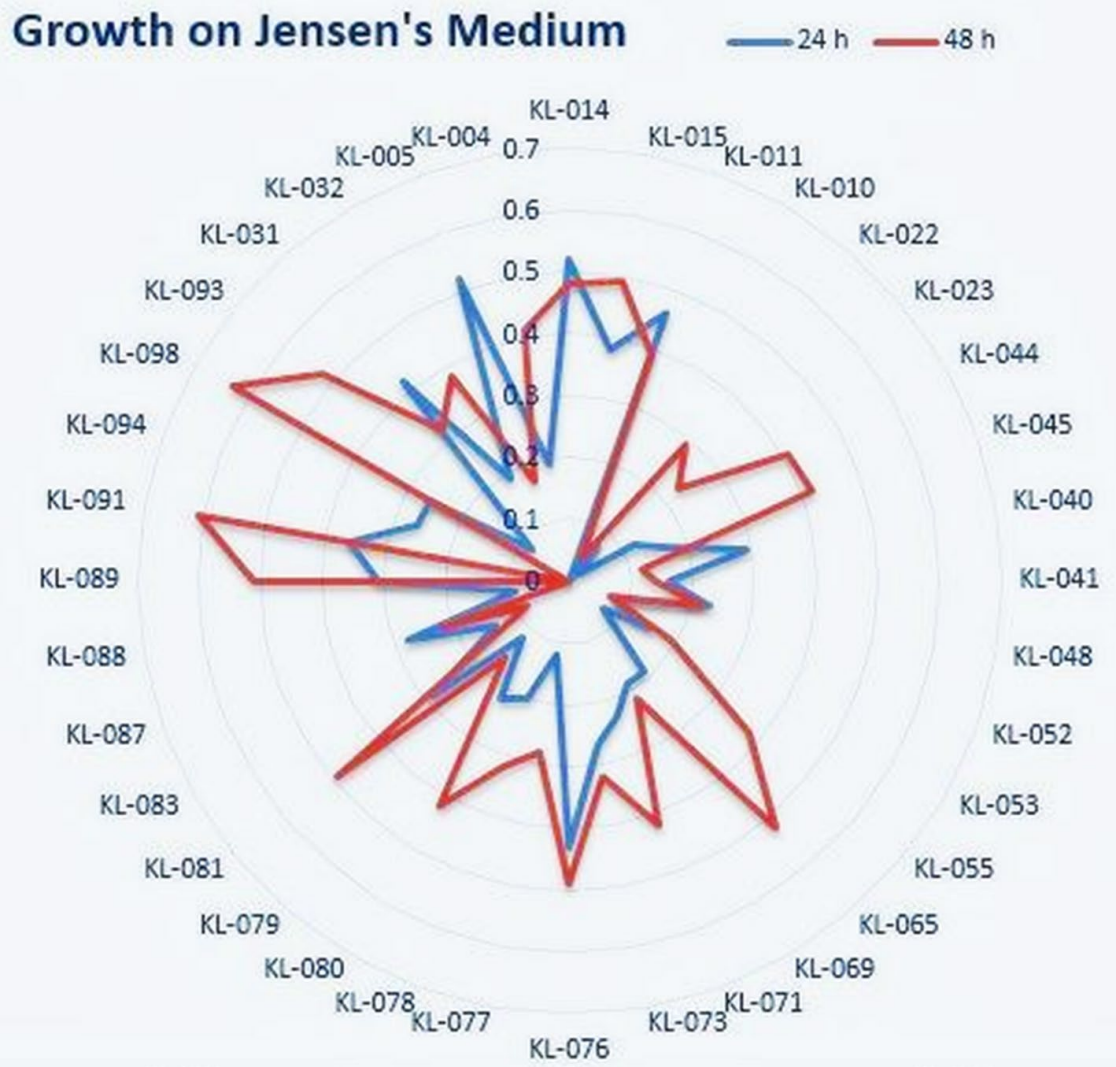
Growth rate on Jensen’s medium

Later, these 36 isolates were subjected to test their nitrogen-fixing capacity. Jensen’s media was used to detect the presence of nitrogen-fixing bacteria. This media was prepared in broth consistency and inoculated with all the selected 36 isolates. All the isolates were grown as triplicates, and the average of all the readings was tabulated as the results in Table 4. As per the table stated below, the isolates KL-014, KL-015, KL-011, KL-076, KL-089, KL-091, and KL-098 showed the best growth on the Jensen’s media by exhibiting their property of nitrogen fixation. These isolates were highlighted in the table. The results were represented in Fig. 4 as the radial picture showing the OD values for both 24 and 48 h in blue and red color respectively.

**Table 4:** Determination of nitrogen-fixing ability

Growth on Jensen’s media (OD_600_)		
Isolate number	**24 h**	**48 h**
KL-014	0.52	0.48
KL-015	0.38	0.49
KL-011	0.46	0.39
KL-010	0	0.04
KL-022	0.07	0.29
KL-023	0.02	0.23
KL-044	0.12	0.41
KL-045	0.16	0.42
KL-040	0.29	0.12
KL-041	0.16	0.14
KL-048	0.23	0.22
KL-052	0.08	0.07
KL-053	0.15	0.19
KL-055	0.07	0.38
KL-065	0.19	0.52
KL-069	0.19	0.22
KL-071	0.23	0.42
KL-073	0.27	0.32
KL-076	0.43	0.49
KL-077	0.12	0.28
KL-078	0.2	0.32
KL-080	0.22	0.42
KL-079	0.12	0.16
KL-081	0.29	0.49
KL-083	0.14	0.08
KL-087	0.28	0.22
KL-088	0.09	0
KL-089	0.31	0.51
KL-091	0.36	0.61
KL-094	0.26	0
KL-098	0.26	0.63
KL-093	0.08	0.52
KL-031	0.42	0.32
KL-032	0.19	0.38
KL-005	0.52	0.17
KL-004	0.19	0.41

**Fig. 5:**
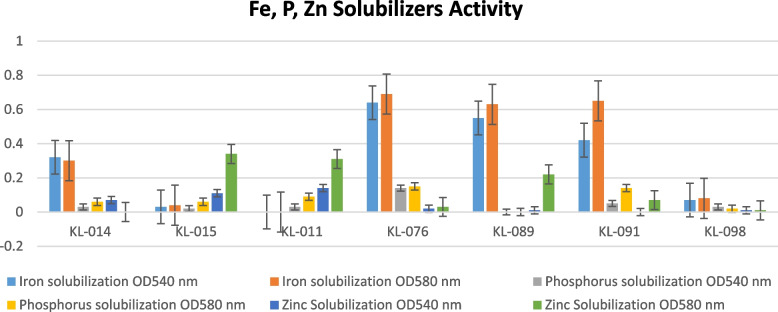
Graphical representation of the isolates representing Fe, P, and Zn activity

The seven best isolates which proved their ability of nitrogen fixation was further moved to examination such as zinc solubilizers, phosphate solubilizers, and zinc solubilizers. The result was tabulated in Table 5 by placing the average of the triplicates of samples along with standard deviation was done. Taking the mean concept and standard deviation into consideration, isolate number KL-076 is best and potential to tolerate different chemical exposures in favor of plant growth-promoting abilities according to Fig. 5. The length of an error bar in a graph serves as a visual indicator of the spread or variability in the data points. Short error bars indicate more confidence in the central value, while long error bars suggest greater uncertainty and less reliability in that value.

**Table 5:** Determination of iron, phosphorous, and zinc solubilization of isolated bacteria

	**Iron solubilizing**			**Phosphorus solubilizing**			**Zinc solubilizing**		
Isolate no	**OD**_**540 nm**_	**OD**_**580 nm**_	**S**	**OD**_**540 nm**_	**OD**_**580 nm**_	**S**	**OD**_**540 nm**_	**OD**_**580 nm**_	**S**
KL-014	0.32	0.3	0.014142	0.03	0.06	0.021213	0.07	0	4.949747
KL-015	0.03	0.04	0.007071	0.02	0.06	0.028284	0.11	0.34	0.162635
KL-011	0	0	0	0.03	0.09	0.042426	0.14	0.31	0.120208
KL-076	0.64	0.69	0.035355	0.14	0.15	0.007071	0.02	0.03	0.007071
KL-089	0.55	0.63	0.056569	0	0	0	0.01	0.22	0.148492
KL-091	0.42	0.65	0.162635	0.05	0.14	0.06364	0	0.07	0.049497
KL-098	0.07	0.08	0.007071	0.03	0.02	0.007071	0.01	0.01	0

**Fig. 6:**
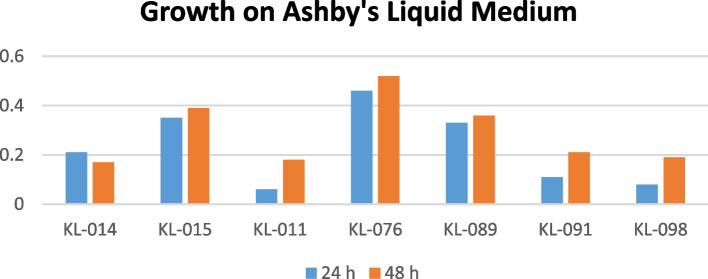
Graphical representation of isolates growth on Ashby’s liquid medium

The isolates that shown best within chemical analysis tests were further examined using Ashby’s nitrogen liquid medium in order to compare their main purpose to be a plant probiotic. Surprisingly, the isolate nos. KL-015, KL-076, and KL-089 have shown positive results indicating their potentiality for nitrogen fixation. Along with these isolates, remaining screened out isolates were subjected to the exposure to liquid medium of Ashby’s. The isolate which had shown growth in liquid medium (Table 6) also was considered as the best one to fulfil the criteria of a nitrogen fixer. The OD value was noted considering the triplicates for each isolate so as to avoid errors. According to Fig. 6, the isolate KL-076 have shown their best growth with Ashby’s liquid medium when compared to other microbial isolates.

**Table 6:** Growth on Ashby’s liquid medium

Growth in Ashby’s liquid Medium (OD_600_)			
Isolate no	**24 h**	**48 h**	**Avg**
KL-014	0.21	0.17	0.19
KL-015	0.35	0.39	0.37
KL-011	0.06	0.18	0.12
KL-076	0.46	0.52	0.49
KL-089	0.33	0.36	0.395
KL-091	0.11	0.21	0.16
KL-098	0.08	0.19	0.135

**Fig. 7:**
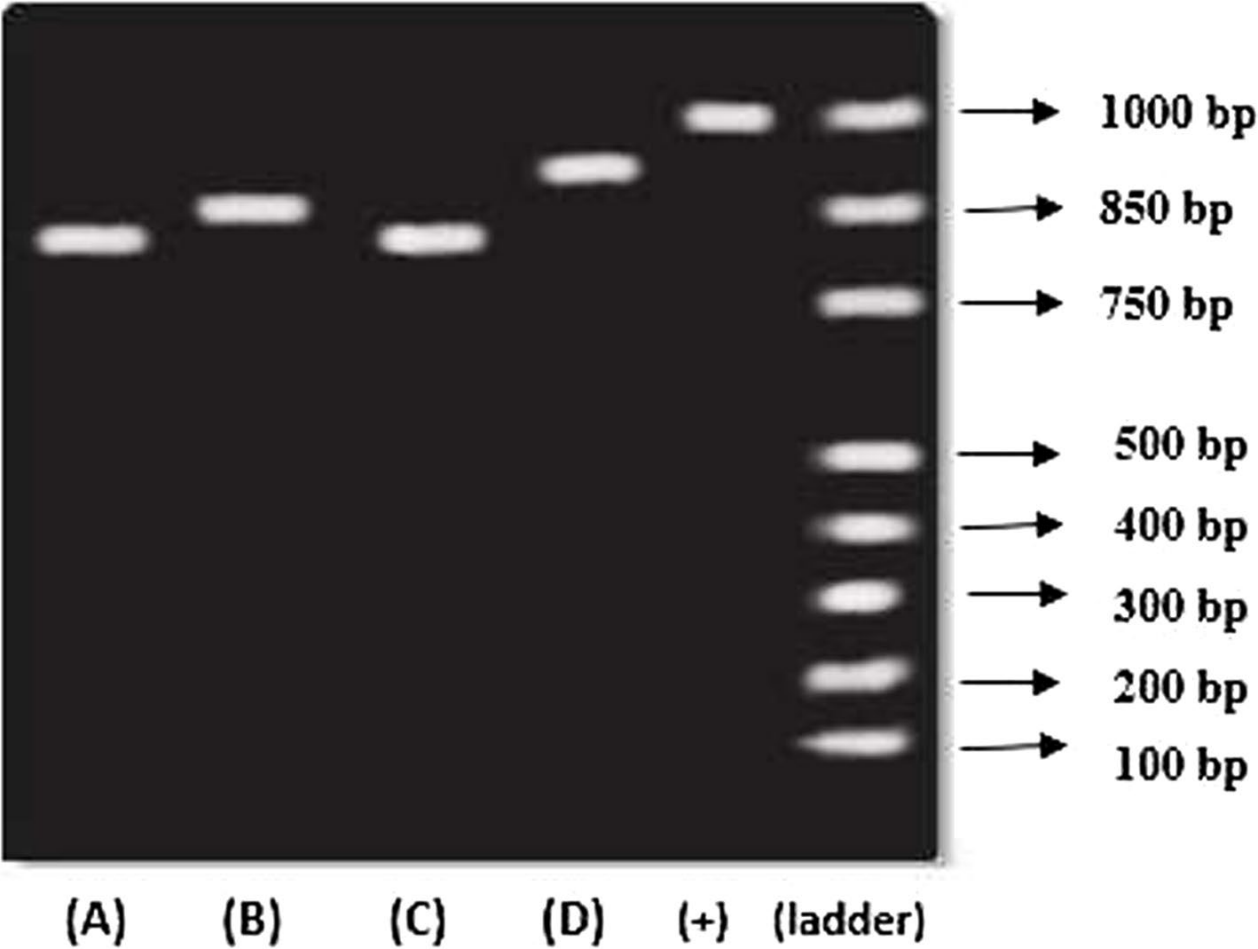
16S rRNA gene amplification obtained from selected bacterial isolates—agarose gel electrophoresis showing amplicons. (1) KL011, (2) KL015, (3) KL076, (4) KL089, (5) positive control, (6) 1-kb DNA ladder

The DNA was analyzed using agarose gel electrophoresis (AGE) technique. Based on the gel amplicon picture, the isolates have shown the bands at app. 1000 kb (Fig. 7). The positive control used in the experiment is 16S gene of a bacteria. The sequence obtained was subjected to BLAST, and further results were tabulated below in Table 7. The table shows the isolated bacteria are closely associated with the corresponding sequences as per the BLAST.

**Table 7:** BLAST results

Label of the isolate	BLASTn result	Accession number of the first organism from BLAST
KL-011	*Corynebacterium accolens*	NR_042139.1
KL-015	*Bacillus rugosus*	NR_181236.1
KL-076	*Lactobacillus pasteurii DSM 23907*	NR_117058.1
KL-089	*Cytobacillus firmus*	NR_112635.1

### 16S sequence obtained for sample KL-011

CTGCACTTCGGGATAAGCTTGGGAAACTGGGTCTAATACCGGATAGGAACCATCTTTAGTGTGATGGTTGGAAAGTTTTTTCGGTGTAGGATGAGCTCGCGGCCTATCAGCTTGTTGGTGGGGTAATGGCCTACCAAGGCGGCGACGGGTAGCCGGCCTGAGAGGGTGTACGGCCACATTGGGACTGAGATACGGCCCAGACTCCTACGGGAGGCAGCAGTGGGGAATATTGCACAATGGGCGCAAGCCTGATGCAGCGACGCCGCGTGGGGGATGAAGGCCTTCGGGTTGTAAACTCCTTTCGCTAGGGACGAAGCTTTTTGTGACGGTACCTAGATAAGAAGCACCGGCTAACTACGTGCCAGCAGCCGCGGTAATACGTAGGGTGCGAGCGTTGTCCGGAATTACTGGGCGTAAAGGGCTCGTAGGTGGTTTGTCGCGTCGTCTGTGAAATTCTGGGGCTTAACTCCGGGCGTGCAGGCGATACGGGCATAACTTGAGTGCTGTAGGGGTAACTGGAATTCCTGGTGTAGCGGTGAAATGCGCAGATATCAGGAGGAACACCGATGGCGAAGGCAGGTTACTGGGCAGTTACTGACGCTGAGGAGCGAAAGCATGGGTAGCGAACAGGATTAGATACCCTGGTAGTCCATGCCGTAAACGGTGGGCGCTAGGTGTGAGGGTCTTTCTACGACTTTCGTGCCGTAGCTAACGCATTAAGCGCCCCGCCTGGGGAGTACGGCCGCAAGGCTAAAACTCAAAGGAATTGACGGGG.

### 16S sequence obtained for sample KL-015

AGCGGTGAAATGCGTAGAGATGTGGAGGAACACCAGTGGCGAAGGCGACTCTCTGGTCTGTAACTGACGCTGAGGAGCGAAAGCGTGGGGAGCGAACAGGATTAGATACCCTGGTAGTCCACGCCGTAAACGATGAGTGCTAAGTGTTAGGGGGTTTCCGCCCCTTAGTGCTGCAGCTAACGCATTAAGCACTCCGCCTGGGGAGTACGGTCGCAAGACTGAAACTCAAAGGAATTGACGGGGGCCCGCACAAGCGGTGGAGCATGTGGTTTAATTCGAAGCAACGCGAAGAACCTTACCAGGTCTTGACATCCTCTGACAATCCTAGAGATAGGACGTCCCCTTCGGGGGCAGAGTGACAGGTGGTGCATGGTTGTCGTCAGCTCGTGTCGTGAGATGTTGGGTTAAGTCCCGCAACGAGCGCAACCCTTGATCTTAGTTGCCAGCATTCAGTTGGGCACTCTAAGGTGACTGCCGGTGACAAACCGGAGGAAGGTGGGGATGACGTCAAATCATCATGCCCCTTATGACCTGGGCTACACACGTGCTACAATGGACAGAACAAAGGGCAGCGAAACCGCGAGGTTAAGCCAATCCCACAAATCTGTTCTCAGTTCGGATCGCAGTCTGCAACTCGACTGCGTGAAGCTGGAATCGCTAGTAATCGCGGATCAGCATGCCGCGGTGAATACGTTCCCGGGCCTTGTACACACCGCCCGTCACACCACGAGAGTTTGTAACACCCGAAGTCGGTGAGGTAACCTTTTAGGAGCCAGCCGCCGAAGGTGGGACAGATGATTGGGGTGAAGTCGTAACAAGGTAGCCGTATC.

### 16S sequence obtained for sample KL-076

ACCCGCGGTGCATTAGCTAGTTGGTAGGGTAAAGGCCTACCAAGGCATTGATGCATAGCCGAGTTGAGAGACTGATCGGCCACATTGGGACTGAGACACGGCCCAAACTCCTACGGGAGGCAGCAGTAGGGAATCTTCCACAATGGACGAAAGTCTGATGGAGCAACGCCGCGTGAGTGAAGAAGGTTTTCGGATCGTAAAGCTCTGTTGTTGGTGAAGAAAGATAGAGAGAGTAACTGATCTTTATTTGACGGTAATCAACCAGAAAGTCACGGCTAACTACGTGCCAGCAGCCGCGGTAATACGTAGGTGGCAAGCGTTGTCCGGATTTATTGGGCGTAAAGCGAGCGCAGGCGGAAAGATAAGTCTGATGTGAAAGCCCTCGGCTCAACCGAGGAACTGCATCGGAAACTGTCTTTCTTGAGTGCAGAAGAGGAGAGTGGAACTCCATGTGTAGCGGTGGAATGCGTAGATATATGGAAGAACACCAGTGGCGAAAGCGGCTCTCTGGTCTGCAACTGACGCTGAGGCTCGAAAGCATGGGTAGCGAACAGGATTAGATACCCTGGTAGTCCATGCCGTAAACGATGAGTGCTAAGTGTTGGGAGGTTTCCGCCTCTCAGTGCTGCAGCTAACGCATTAAGCACTCCGCCTGGGGAGTACGACCGCAAGGTTGAAACTCAAAGGAATTGACGGGGGCCCGCACAAGCGGTGGAGCATGTGGTTTAATTCGAAGCAACGCGAAGAACCTTACCAGGTCTTGACATCTAGCGCAATCCCAAGAGATTGGGAGTTCCCTTCGGGGACGCTAAGACAGG.

### 16S sequence obtained for sample KL-089

GGGTGATCGGCCACACTGGGACTGAGACACGGCCCAGACTCCTACGGGAGGCAGCAGTAGGGAATCTTCCGCAATGGACGAAAGTCTGACGGAGCAACGCCGCGTGAGTGATGAAGGTTTTCGGATCGTAAAACTCTGTTGTCAGGGAAGAACAAGTACCGGAGTAACTGCCGGTACCTTGACGGTACCTGACCAGAAAGCCACGGCTAACTACGTGCCAGCAGCCGCGGTAATACGTAGGTGGCAAGCGTTGTCCGGAATTATTGGGCGTAAAGCGCGCGCAGGCGGTTCCTTAAGTCTGATGTGAAAGCCCCCGGCTCAACCGGGGAGGGTCATTGGAAACTGGGGAACTTGAGTGCAGAAGAGAAGAGTGGAATTCCACGTGTAGCGGTGAAATGCGTAGAGATGTGGAGGAACACCAGTGGCGAAGGCGACTCTTTGGTCTGTAACTGACGCTGAGGCGCGAAAGCGTGGGGAGCAAACAGGATTAGATACCCTGGTAGTCCACGCCGTAAACGATGAGTGCTAAGTGTTAGAGGGTTTCCGCCCTTTAGTGCTGCAGCAAACGCATTAAGCACTCCGCCTGGGGAGTACGGCCGCAAGGCTGAAACTCAAAGGAATTGACGGGGGCCCGCACAAGCGGTGGAGCATGTGGTTTAATTCGAAGCAACGCGAAGAACCTTACCAGGTCTTGACATCTCCTGACAACCCTAGAGATAGGGCGTTCCCCTTCGGGGGACAGGATGACAGGTGGTGCATGGTTGTCGTCAGCTCGTGTCGTGAGATGTTGGGTTAAGTCCCGCAACGAGCGCAACCCTTGATCTTAGTTGCCAGCATTCAGTTGGGCACTCTAAGGTGACTGCCGGTGACAAACCGGAGGAAGGTGGGGATGACGTCAAATCATCATGCCCCTTATGACCTGGGCTACACACGTGCTACAATGGATGGTACAAAGGGCTGCAA.

**Fig. 8:**
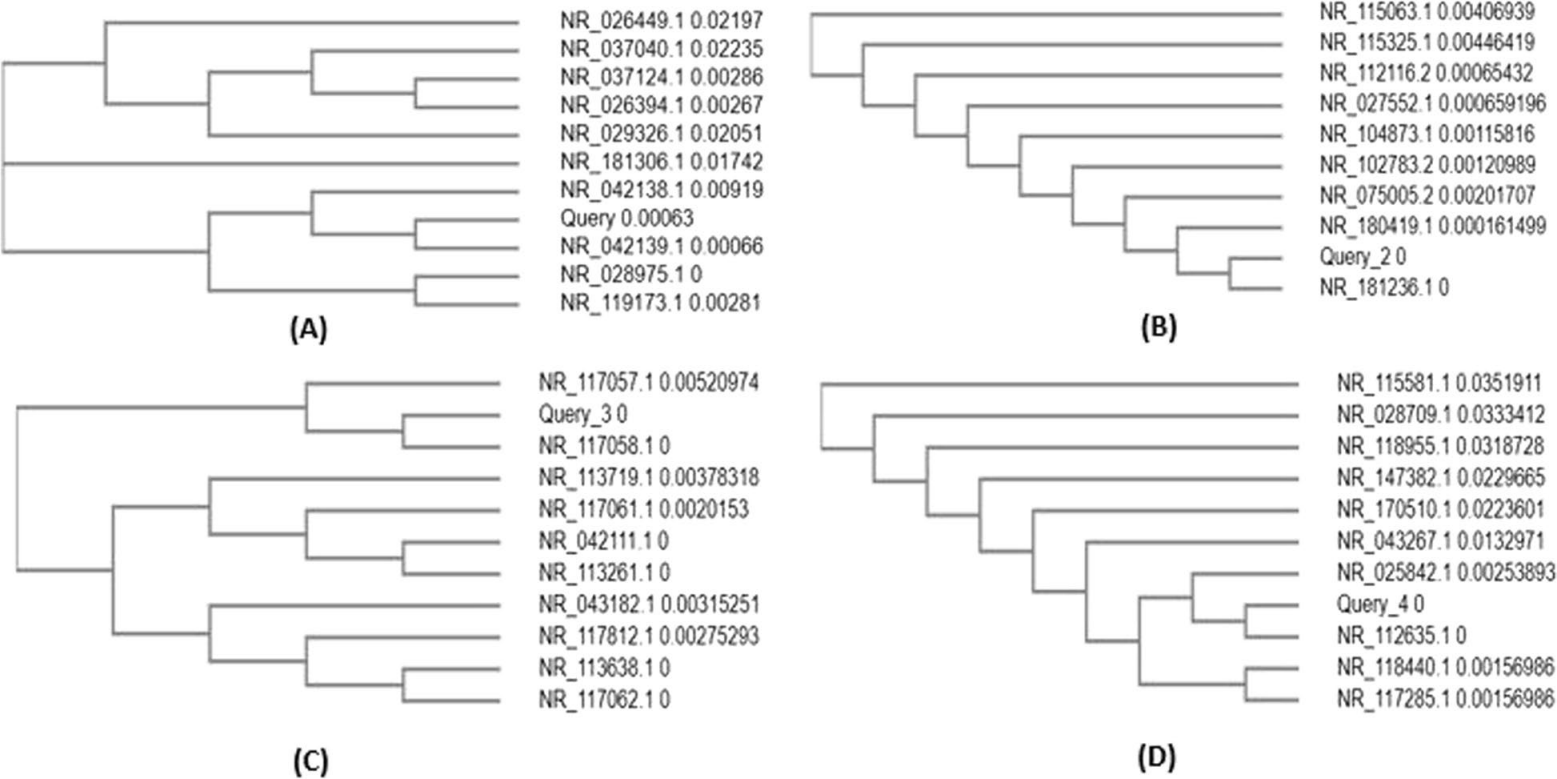
Cladogram of **A** KL-011, **B** KL-015, **C** KL-076, **D** KL-089

**Fig. 9:**
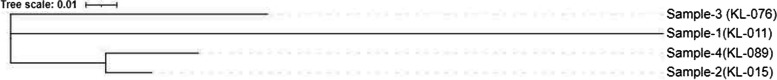
Bootstrapping results using IQ-TREE

The above sequences were used to run the BLAST separately, and later on, the top 10 sequences were used to construct the cladogram using Clustal Omega tool as shown in Fig. 8. The query in the cladogram shows its branch connecting to the closely associated bacterial strain. The results were coincided with the strain shown in the BLAST results. To increase the algorithmic accuracy of the inferred phylogenetic trees, bootstrapping is performed which is represented in Fig. 9. It constructs multiple phylogenetic solutions for a dataset, and the consistent scores of various branches and the placement of their nodes are assessed to statistically screen the most accurate solution.

## Discussion

To select a bacteria as a potent plant probiotic, it must be beneficiary to humans also as the end result of this concept is growth of a healthy plant. As we know, the greatest ecological and agronomical advantages derived by plants from their contact with microbes are biological nitrogen fixation by bacteria. It is believed that the bacterial isolates which were finally identified within this work are the one which can convert the atmospheric nitrogen to ammonia using its nitrogenase enzyme, thus making it available for plant absorption. The presence of the nitrogenase enzyme decides the plant’s maturity and adds economic value too. The bacteria may also be capable to utilize various chemicals such as zinc which stimulates enzymes that are involved in the creation of some proteins. It aids in the production of chlorophyll and certain carbohydrates, the transformation of starches to sugars, and its availability in plant tissue aids in the plant’s resistance to low temperatures [43]. Zinc is required to produce auxins, aiding growth control and stem elongation. On the other context, the higher concentrations of the same can be toxic to the plant and therefore require a limited supply of zinc to maintain the optimal concentration in the plant [44]. Iron is the most prioritized element for a plant without which it cannot synthesize chlorophyll. Many of the plant metabolic pathways are activated by iron behaving as the main cofactor of the majority of key enzymes [45]. Iron is mainly absorbed by the plant and solubilizes ferric to reduce to ferrous which could be a convenient route for transportation. Because of this role, the media was also modified in a way that has enough volume of ferric chloride in it. Some bacteria produce iron-chelating compounds known as siderophores. The bacteria specifically recognize and sequester the limited supply of iron in the rhizosphere. This process reduces the availability of iron to pathogenic microbes and simultaneously supplies iron to the plant [46]. According to recent studies, phosphorus is one of the most recommended chemicals used in the manufacturing of fertilizers which plays a key role in enhancing plant growth. Plants absorb phosphorus in ionic form such as dihydrogen orthophosphate or monohydrogen diphoshate [47]. Plants require proper amounts of phosphorus for optimal development. It involves nearly every major metabolic function, including energy transmission, signalling, metabolism, complex molecular biosynthesis, and photosynthesis [48]. Generally, the phosphorus present in the soil forms an aggregate with other chemicals. The phosphate-solubilizing bacteria breaks up such bonds and makes the phosphorus available for the plant [49]. As a component of the identification process, the agarose gel electrophoresis (AGE) technique was executed. AGE stands out as a versatile and extensively employed method in molecular biology due to its simplicity, cost efficiency, and capacity to segregate DNA fragments according to their sizes. This separation enables the derivation of deductions about the samples and facilitates subsequent downstream analyses. Ultimately, the isolated bacteria underwent identification through a series of steps including sequence alignments, a database search using BLASTn, phylogenetic analysis, and correlation. Based on the findings, several strains have displayed resemblances in terms of either query cover or percentile identity. However, these resemblances are influenced by limitations that impede the technique from providing unequivocal identification of distinct strains or species. These limitations encompass confined resolution, sequence variability, intraspecies disparities, restrictions within reference databases, and the potential implications of distinct primer sets. These sets can differ in terms of their precision and scope.

## Conclusion

“Plant probiotics” is an emerging and intriguing concept within the realm of agriculture and plant science. This concept revolves around the idea of using beneficial microorganisms to enhance plant health and growth. This innovative approach delves into the analysis of plant immunity as well, aiming to better understand how these microorganisms can interact with plants’ natural defense mechanisms. Numerous studies have drawn attention to the significant role that plant probiotics can play. These studies have identified specific microorganism isolates that have demonstrated particularly promising results. These promising isolates are then subjected to a thorough screening process to assess their potential. This assessment involves employing techniques such as biopriming, which entails treating seeds with these beneficial microorganisms to facilitate their growth and establishment in the plant’s environment. Moreover, we are simultaneously working on optimizing the growth conditions and parameters for these beneficial microorganisms. This involves creating an environment that allows these microorganisms to thrive and interact harmoniously with the plant. The ultimate goal is to develop a technique that can reliably enhance plant health and performance. One aspect of research in this area focuses on investigating the specific compositions of microbial communities in the soil. Different combinations of microorganisms can have varying effects on soil fertility and subsequently impact the development of plants that grow in that soil. Through careful analysis, researchers seek to uncover the intricate relationships between these microorganisms and the overall health of both the soil and the plants. Early findings suggest that these microbial communities possess an adaptive capability, allowing them to adjust and thrive under different chemical conditions present in the soil. This adaptability opens up avenues to cultivate these microorganisms into potent plant probiotics that can potentially withstand a range of environmental challenges. In summary, the emerging field of plant probiotics holds immense promise for revolutionizing agriculture and plant cultivation.

### Supplementary Information

**Additional file 1.** BLASTN Results.

## Data Availability

Not applicable.
