# Supplementary figures and images for: Isolation of plant growth-promoting rhizobacteria from the agricultural fields of Tattiannaram, Telangana
Source: J Genet Eng Biotechnol. 2023 Dec 6;21:159. doi: 10.1186/s43141-023-00615-5 (PMC10697926; doi:10.1186/s43141-023-00615-5)

**BLASTN Results**

**Sample -1 (KL-011)**


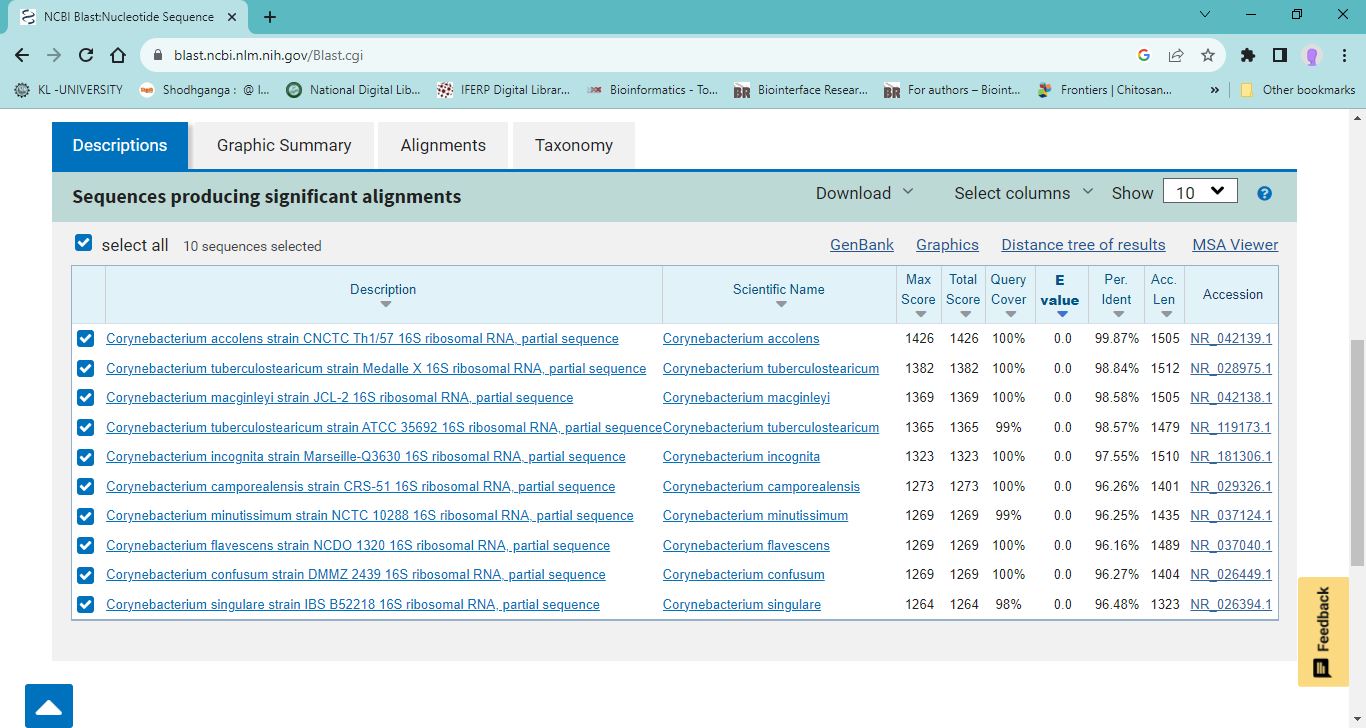


**Sample -2 (KL-015)**


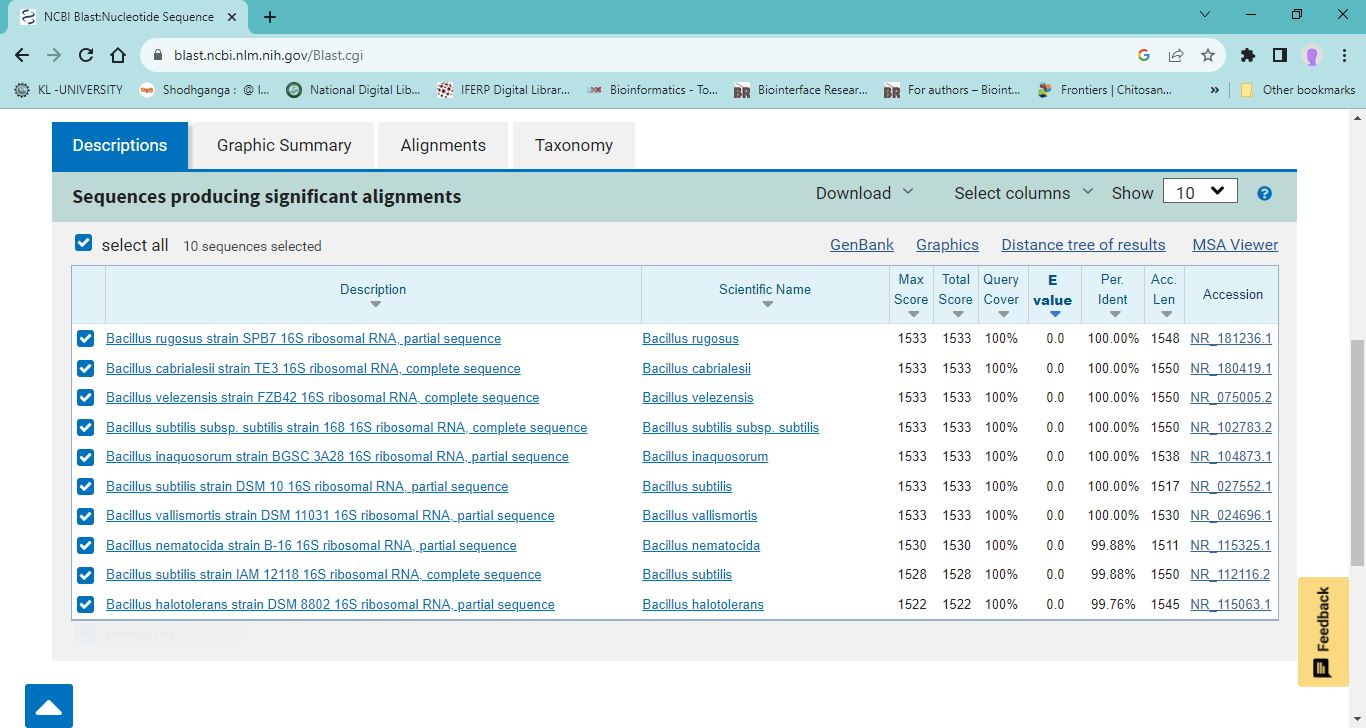


**Sample -3 (KL-076)**


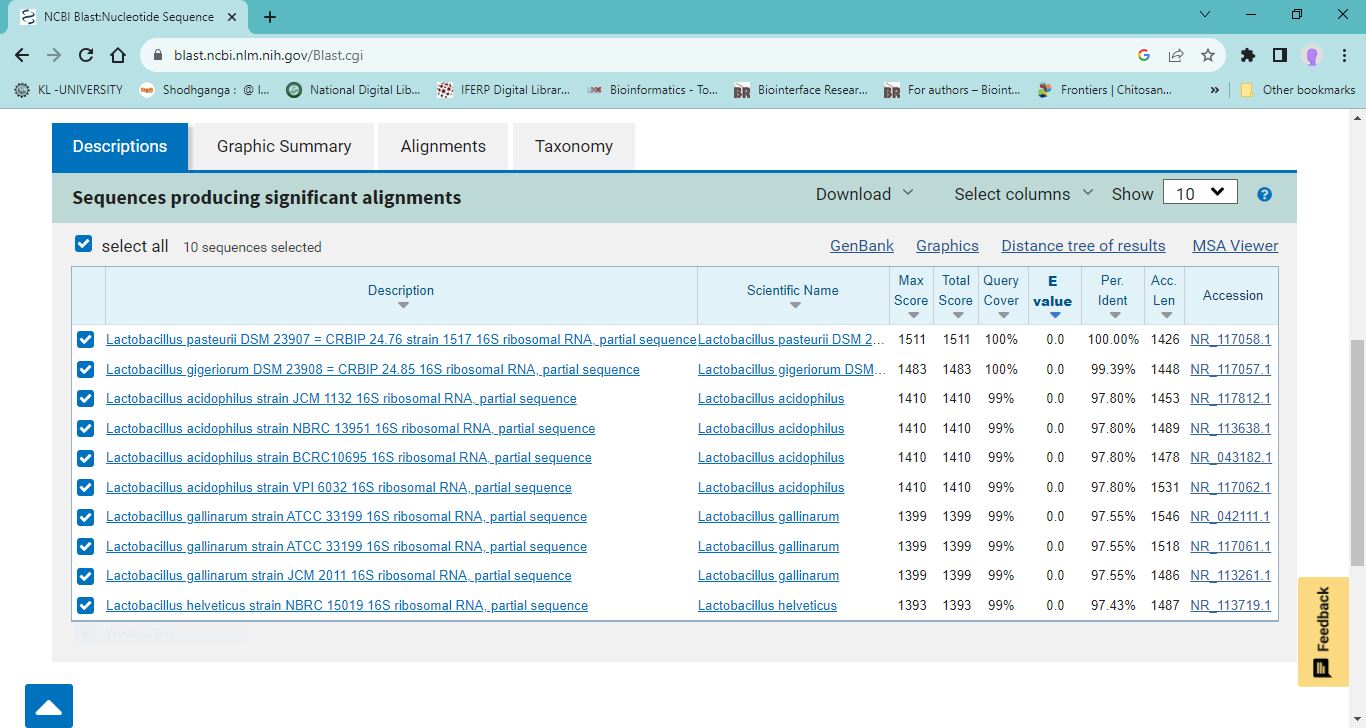


**Sample -4 (KL-089)**


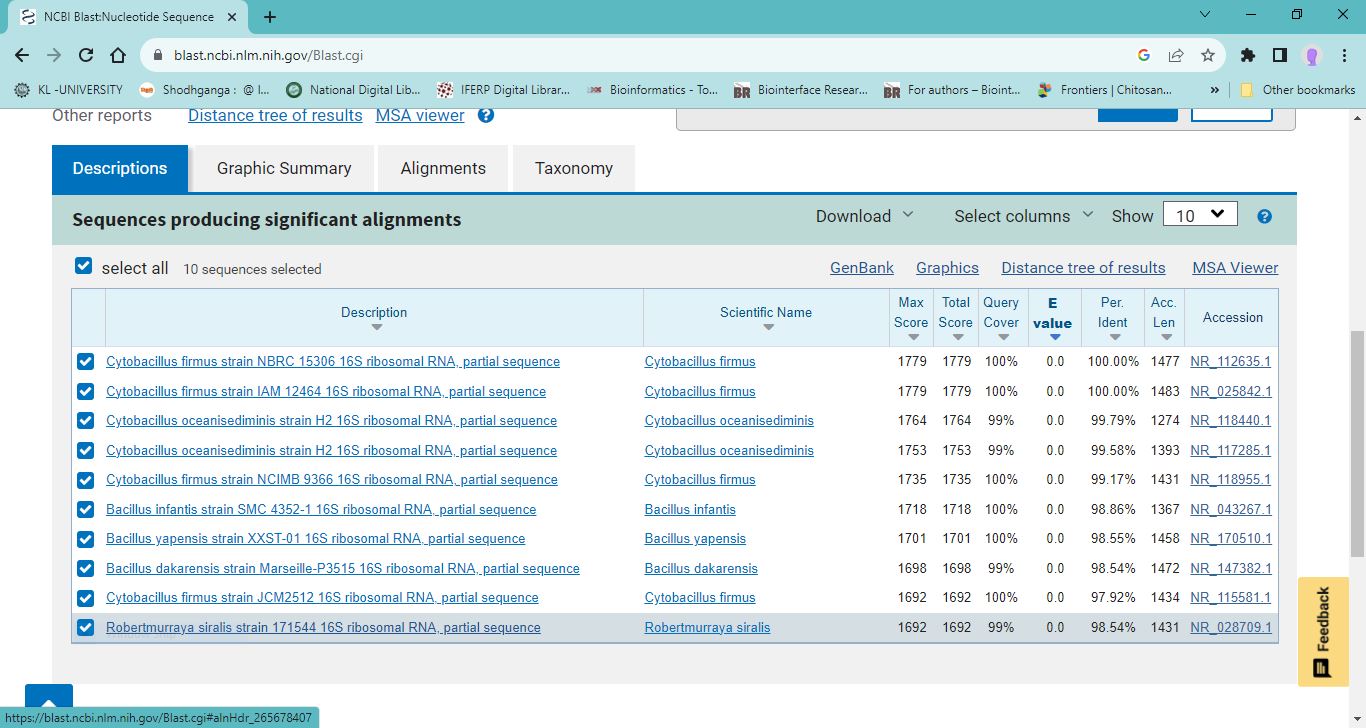

Supplement: Supplementary file 1 — Additional file 1. BLASTN Results. [file 43141_2023_615_MOESM1_ESM.docx]
